# On the correlation between microscopic structural heterogeneity and embrittlement behavior in metallic glasses

**DOI:** 10.1038/srep14786

**Published:** 2015-10-05

**Authors:** Weidong Li, Yanfei Gao, Hongbin Bei

**Affiliations:** 1 Department of Materials Science and Engineering, University of Tennessee, Knoxville, TN 37996; 2 Materials Science and Technology Division, Oak Ridge National Laboratory, Oak Ridge, TN 37831.

## Abstract

In order to establish a relationship between microstructure and mechanical properties, we systematically annealed a Zr-based bulk metallic glass (BMG) at 100 ~ 300 °C and measured their mechanical and thermal properties. The as-cast BMG exhibits some ductility, while the increase of annealing temperature and time leads to the transition to a brittle behavior that can reach nearly-zero fracture energy. The differential scanning calorimetry did not find any significant changes in crystallization temperature and enthalpy, indicating that the materials still remained fully amorphous. Elastic constants measured by ultrasonic technique vary only slightly with respect to annealing temperature and time, which does obey the empirical relationship between Poisson’s ratio and fracture behavior. Nanoindentation pop-in tests were conducted, from which the pop-in strength mapping provides a “mechanical probe” of the microscopic structural heterogeneities in these metallic glasses. Based on stochastically statistic defect model, we found that the defect density decreases with increasing annealing temperature and annealing time and is exponentially related to the fracture energy. A ductile-versus-brittle behavior (DBB) model based on the structural heterogeneity is developed to identify the physical origins of the embrittlement behavior through the interactions between these defects and crack tip.

Metallic glasses, or called amorphous alloys, have attracted considerable interests due to their unique mechanical properties for potential structural applications^1^,^2^,^3^. The deformation behavior of these materials usually involves strain localization in narrow shear bands, which, if unconstrained, can easily lead to catastrophic failure. Avoiding catastrophic failure and designing extraordinarily ductile metallic glasses has clearly been a constant pursuit of numerous studies. A variety of methods have been developed to block the propagation of these shear bands, so that the applied strain can be equally accommodated by a few of shear bands and thus the failure at the shear bands will be delayed. Since the shear band direction is usually very close to the principal shear stress direction, shear bands in uniaxially compressed samples with low height-to-diameter ratios can be stopped at the sample-platen interface, and thus enhanced ductility has been found^4^. A similar line of argument also applies for metallic glass coatings^5^,^6^, composites^7^,^8^, and other types of geometric constraints. Similar to the tempered window glass, one can introduce residual stress to prevent the shear band initiation and propagation and thus to enhance the hardness^9^,^10^. Besides these above-mentioned *extrinsic* approaches, the *intrinsic* ductile/brittle behavior of metallic glasses has been extensively studied by fracture toughness measurements. The measured toughness varies significantly from a few MPa·m^1/2^ (brittle) to hundreds of MPa·m^1/2^ (ductile), depending on composition, processing history like annealing and cooling rate during solidification^11^,^12^, or internal structural variation in terms of free volume, shear transformation zone (STZ) or atomic configurations^13^,^14^,^15^. For example, Mg_65_Cu_25_Tb_10_ metallic glass fractures at nearly zero plasticity with a fracture toughness of ~2 MPa·m^1/2^^16^. But for some others such as Zr_41.25_Ti_13.75_Ni_10_Cu_12.5_Be_22.5_ metallic glass, the fracture toughness can be ~130 MPa·m^1/2^ 17,18. For the same metallic glass, thermal treatment like annealing may change the toughness by several orders of magnitude. This has critical implications in applications such as the use of Fe-based metallic glass wires in transformers, which are subject to Joule heating and thus various heating/cooling histories. Obviously a systematic change of time and temperature, together with the investigation of the ductile-versus-brittle behavior (DBB), will help identify the key factors that eventually lead to the development of “immortal” metallic glasses for such applications as in transformers. This line of research will also help reduce the testing time since the actual applications are operated in a time scale not achievable in laboratory tests. It should be noted that we prefer not to use the term of ductile-to-brittle transition (DBT) here because the thermal treatment here changes material microstructure, while DBT typically refers to the change of mechanical properties with respect to temperature but without the change of microstructure.

One effort in the study of intrinsic brittle/ductile behavior of metallic glasses is to correlate the fracture toughness to Poisson’s ratio, 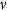, or equivalently the shear-to-bulk modulus ratio 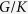
19,20,21,22,23. By compiling experimental data, it is suggested that Poisson’s ratio in excess of 0.31 ~ 0.32 implies good ductility and high fracture toughness. Or equivalently, a small (large) 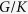 favors high (low) fracture toughness—a critical value of 0.41 ~ 0.43 was suggested. Such a correlation is a loaned concept from crystalline materials, in which Poisson’s ratio is related to the competition between the emission of dislocation and cleavage fracture that scale with shear modulus and bulk modulus, respectively^24^,^25^,^26^. Owing to the absence of dislocation, the above scenario does not apply for metallic glasses, and also the above empirical relationship is found not to work for some metallic glasses such as the Pd-based ones^27^,^28^. Since the deformation behavior of amorphous alloys relies on a number of factors such as composition and processing history, it appears to be inappropriate for the DBB to be governed by an elastic parameter.

From the micromechanical point of view, the fracture toughness variation depends on the competition of two or many mechanisms – being dislocation emission and cleavage in single crystals. Attempts have been made to rationalize the DBB behavior in metallic glasses along this line, e.g., by the competition between cleavage fracture and shear band initiation from the crack tip. Poon *et al.*^21^ argued that a large Poisson’s ratio requires a large mechanical energy that needs to activate a shear transformation zone through an Eshelby-type transformation process, so that large fracture energy is required to initiate shear bands at the crack tip. Based on the Spaepen’s free volume model^29^,^30^,^31^, which models the shear banding process, and the cohesive interface model, which represents the cleavage fracture process, our finite element simulations have found rather complicated, non-monotonic dependence on Poisson’s ratio. First, the stress fields near a crack tip may change considerably with respect to Poisson’s ratio, especially when a large degree of mode mixity (Mode I versus Mode II) is introduced. Second, the dependence of shear banding behavior on Poisson’s ratio is poorly understood. The Spaepen model only considers the shear-driven free volume evolution process, while hydrostatic stress can also lead to dilatation, free volume change, and thus a complicated dependence on Poisson’s ratio. Unfortunately, none of the above processes can be easily examined by microstructural characterization methods.

It is now well accepted that the microstructure of metallic glasses is intrinsically heterogeneous with the atomic configurations (or conceptually free volume or STZ) varying from sites to sites^32^,^33^,^34^. Murali *et al.*^35^,^36^ used atomistic simulations to confirm that the spatial fluctuations of local material properties control the brittle or ductile fracture of a metallic glass. A high degree of atomic spatial fluctuations leads to nanoscale cavitation, and these cavities will grow and easily coalescence into a brittle crack. However, it should be noted that a large fracture toughness and good ductility should be a result of an energy dissipating zone in the vicinity of the crack tip by many shear bands. In other words, whether a single shear band is prone to cavitation failure depends on nanoscale structural heterogeneities, but the crack tip blunting should be governed by microscopic structural heterogeneities that dictate the collective shear banding behavior. The counterpart in crystalline materials is that toughening mechanisms are related to the collective behavior of dislocations and microstructures in the crack tip process zone, but not by a single dislocation emission at the crack tip. On the other hand, it is very difficult for microstructural characterization methods such as transmission electron microscopy and synchrotron x-ray diffraction to identify these heterogeneities and their effects on the resulting mechanical properties^37^,^38^. In our recent work^39^, we notice that since the structural heterogeneity is directly linked to fluctuations in mechanical properties, one may characterize the microstructure of a metallic glass by probing its spatial mechanical heterogeneity. The challenge along this line of thought is the length scale – the structural heterogeneity is on or below the micron scale, so that the nanoindentation test is ideal to characterize such a spatial fluctuation.

In this work, we aim to identify the crucial physical factors, particularly the structural heterogeneity, which govern the *intrinsic* ductile/brittle behavior of metallic glasses. The relatively ductile as-cast metallic glass samples were first annealed at a wide range of temperatures (100 ~ 300 °C) and times (1/6 ~ 1444 hours) to change its internal structural heterogeneity. The facture energies were measured by using three point bending and then correlated to Poisson’s ratio 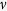, shear-to-bulk modulus ratio 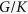, and the thermal properties of the metallic glasses. Statistical nanoindentation test was applied to investigate the mechanical heterogeneity of the as-cast and annealed samples, in order to establish the relation between structural heterogeneity and DBB of BMGs. A DBB model is finally developed based on the interactions between crack tip processes and surrounding microscopic structural heterogeneities.

## Results

### Ductile versus brittle behavior in fracture tests

**Figure 1:**
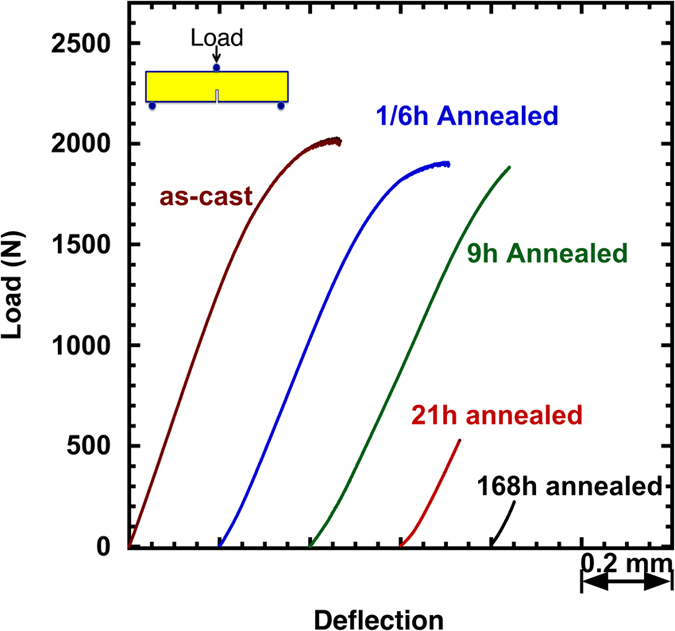
Load-deflection curves for as-cast and annealed (300 °C) Zr-based bulk metallic glass samples obtained from the notched three-point bending tests.

**Figure 2:**
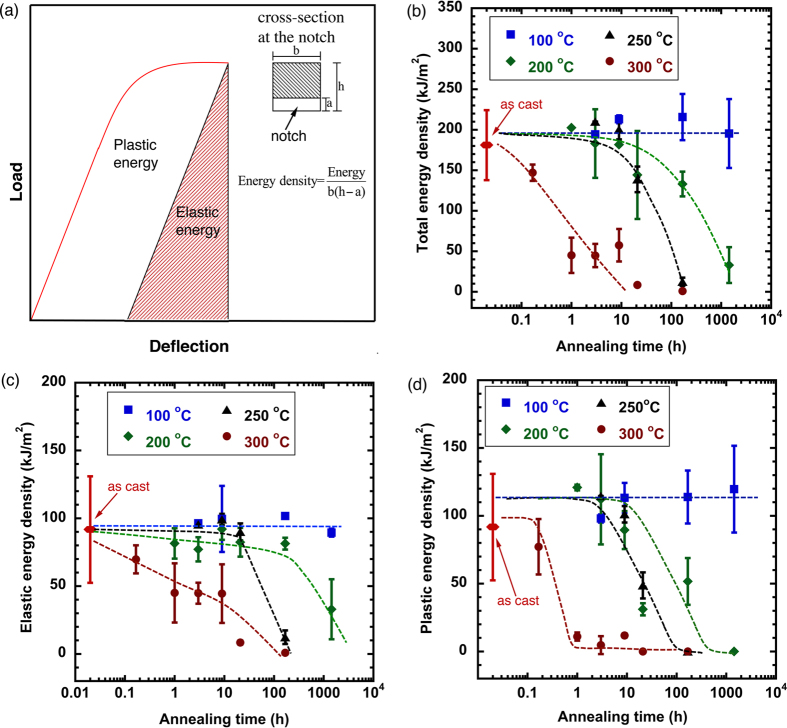
(**a**) Schematic showing how the elastic, plastic and total energy are calculated. These calculated energies are further divided by the unnotched area (inset) to calculate the energy density. Calculated total, elastic and plastic energy densities are given in (**b–d)**, respectively.

Three point bending tests were repeated on variously annealed samples, with annealing temperature and time ranging from 100 °C to 300 °C, and 1/6 hours to 1440 hours. Figure 1 shows the load-deflection curves for samples under as-cast and 300 °C annealed conditions. For the as-cast sample, following a linear load-deflection relation at low loads, a nonlinear portion starts at ~1500 N and a large degree of deflection is reached before failure. Annealed at 300 °C for short times (1/6 and 9 hours), these samples still maintain the nonlinear behavior but its portion decreases with the increase of annealing time. Increasing the annealing time to 21 and 168 hours leads to perfectly brittle failure in elastic region. To obtain quantitative information in ductile versus brittle behavior in the BMG, we measure the fracture energy density, which represent the fracture energy per unit area of the unnotched surface, as defined in the inset in Fig. 2(a). The elastic, plastic and total energies are first calculated by integrating the corresponding areas underneath the load-deflection curves, as schematically shown in Fig. 2(a). The energy density is then obtained by dividing a calculated energy by the corresponding unnotched surface area. Given that all tested samples have almost identical dimensions (2.5 × 2.5 × 15.0 mm), the energy density calculated this way allows a sound comparison of different samples.

The total energy density as a function of the annealing conditions is plotted in Fig. 2(b). Essentially, the energy density maintains at a level of ~200 kJ/m^2^ as the samples are being annealed at 100 °C for up to 1440 hours. No embrittlement occurs. This implies that at this temperature the ductility of the metallic glass is independent of the annealing time. In other words, the metallic glass could never become brittle regardless of the annealing time. Identifying such a critical temperature is of particular interest. For example, when a metallic glass component is designed for high temperature applications, like in transformers, the knowledge of this critical temperature can guarantee the proper operation of the component without temporal degradation. As the annealing temperature goes up to 200 °C, a gradual drop in the fracture energy density starts from the annealing time of 9 hours, indicating a transition from ductile to brittle behavior. Annealing at 250 °C still finds such a ductile-to-brittle transition at 9 hours but the rate of the energy drop increases, and the nearly-zero energy state is rapidly reached (168 hours). This suggests that an increase in the annealing temperature will lead to an earlier ductile-to-brittle transition. Such a trend is further supported by 300 °C annealing condition, in which the fracture energy decrease starts even at 1/6 hours and rapidly drops to almost zero at 1 hour. To this end, increasing the annealing temperature is equivalent to extending the annealing time when the annealing temperature is above the aforementioned critical temperature (~100 °C in this work). Variation of the elastic and plastic energy densities for different annealing temperatures and times shows essentially the same trends in Fig. 2(c,d). It should be noted that the dependence of the ductile versus brittle behavior on annealing conditions have been well documented in literature^40^,^41^,^42^,^43^. The objective here is to provide a quantitative measure of the macroscopic behavior, i.e., the fracture energy in Fig. 2, which will be used to relate to the microscopic measure of structural heterogeneity in later part of this work.

**Figure 3:**
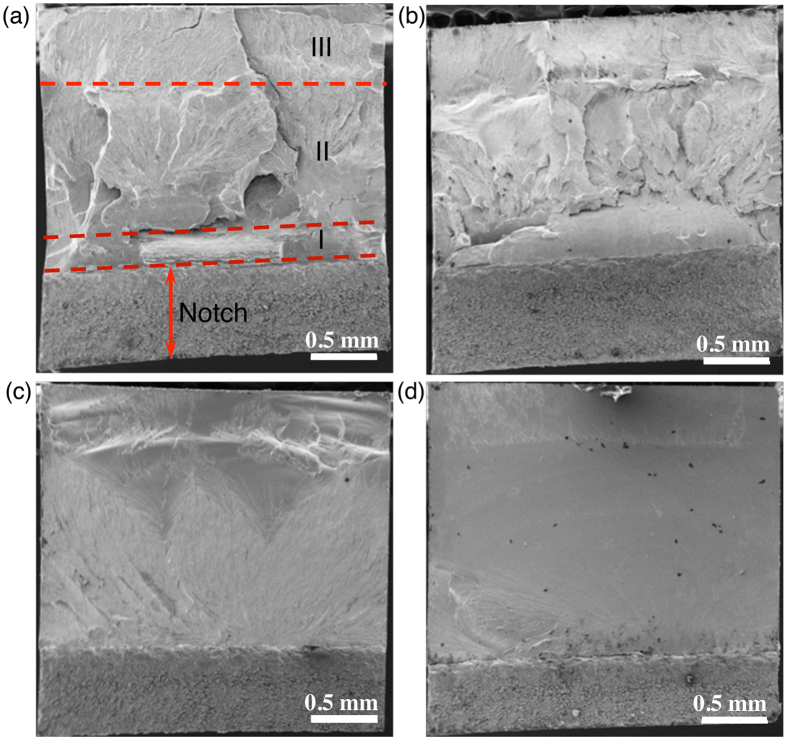
Fracture surfaces of the as-cast sample in (a) and samples annealed at 300 °C for 9 hours in (b), 21 hours in (c) and 168 hours in (d).

The as-cast and 300 °C-annealed samples are selected for fractography study after the bending tests. For the as-cast and 9 hour-annealed sample, since some degrees of plasticity exist (Fig. 2(a)), rough fracture surfaces are found in Fig. 3(a,b). The rough surfaces are formed by the shear band propagation along wavy paths. Close examination finds that fracture of a relatively ductile metallic glass sample consists of three stages. As representatively shown by the as-cast sample in Fig. 3(a), as soon as a sharp crack initiate from the interior end of the notch, it propagates rapidly to a short distance because of sudden release of the stored energy, as indicated by Stage I. Subsequent crack propagation is slow and branches sideward due to the blunting effect of the developed plastic deformation. This forms a largely extended and rather rough fracture region, as indicated by Stage II. With further increase of the external load, the crack propagates rapidly and catastrophically. On the contrary, bending tests on samples annealed for 21 and 168 hours give rise to relatively flat fracture surfaces, as demonstrated in Fig. 3(c,d). Particularly, fracture surface of the 168 hour-annealed sample exhibits a mirror finish. In these two cases, the intrinsic plasticity has little resistance to the crack propagation, and cracks will propagate rapidly throughout the entire sample once initiated.

**Figure 4:**
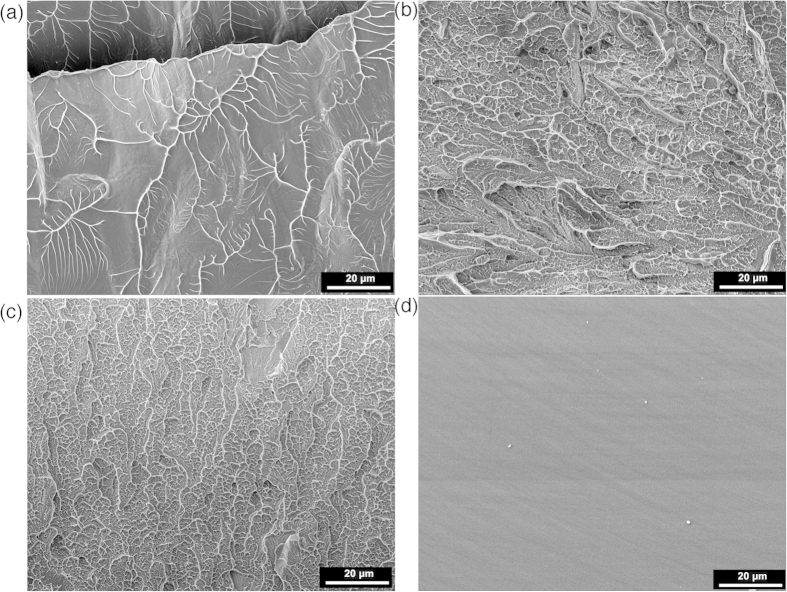
Fractography of the as-cast sample in (a) and samples annealed at 300 °C for 9 hours in (b), 21 hours in (c) and 168 hours in (d) at high magnification, showing the transition from vein patterns, to dimples, and to mirror-like smooth surface.

Higher resolution images on the fracture surfaces show typical vein patterns for the as-cast sample in Fig. 4(a), dimples in the 21-hour annealed sample in Fig. 4(c), and the mixed scenario in the 9-hour annealed sample in Fig. 4(b). No visible features are present in the 168-hour annealed sample in Fig. 4 (d). The general tendency for the variation of the vein pattern is that its size decreases with increasing annealing time until it vanishes at a certain condition. Vein pattern is often a result of fluid meniscus instability mechanism in ductile fracture, while dimples are due to the nanoscale cavitation in brittle fracture in metallic glasses^35^,^36^.

### Correlation between DBB and thermal properties

**Figure 5:**
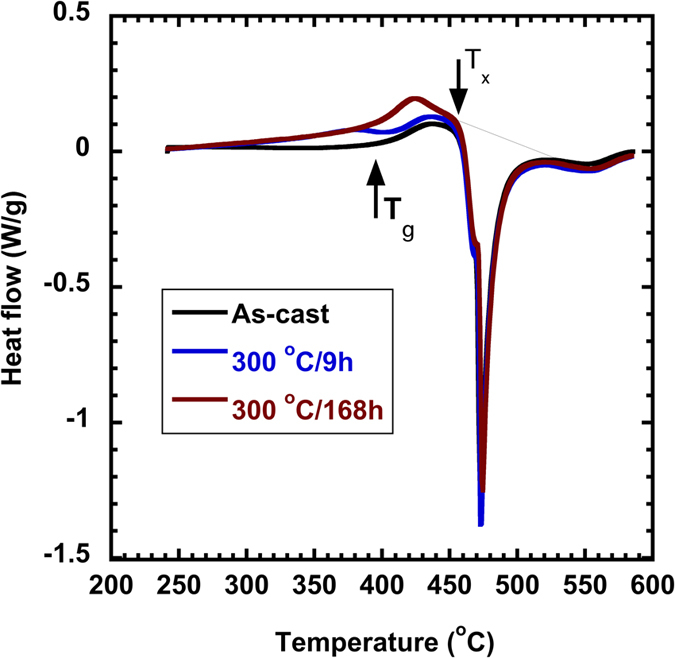
DSC traces of the as-cast and annealed samples with two different annealing durations.

Figure 5 shows the DSC heating curves for as-cast, 300 °C-9 hour and 300 °C-168 hour annealed BMG samples from ~250 to 600 °C. Glass transition and crystallization (exothermal peak) can be clearly seen in all samples, which can be used to determine the glass transition temperature (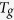, upper arrow in Fig. 5), the onset of crystallization temperature (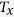, lower arrow in Fig. 5), the supercooled liquid region 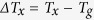, and the enthalpy of crystallization (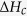). As sown in Table 1, the onset of crystallization temperature and the enthalpy of crystallization are almost identical within experimental accuracy, indicating that the samples are still fully amorphous. This is understandable because the highest annealing temperature we have selected is still far below 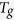 and 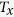 Therefore we can rule out the possibility of nanocrystallization-induced embrittlement.

In Table 1, 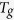 for annealed samples are 406 °C and 405 °C respectively, slightly larger than 398 °C of the as-cast sample. Accordingly, 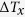 for the two annealed samples (52.5 °C, 54.0 °C) are slightly narrower than that of the as-cast sample (61.0 °C). The glass transition behavior reflects the atomic transport and viscosity properties which dominate the processes of glass forming, structural relaxation and thermal stability in the amorphous structure^44^,^45^,^46^. A slight increase in *T*_*g*_, or decrease in *∆T*_*x*_ in annealed samples, or shape changes in the glass transition peaks do reflect a certain degree of structural relaxation upon annealing. However, glass transition is not a first-order phase transformation (e.g., the heat generated/absorbed is very small), these slight changes are difficult to quantify the structure-mechanical property relationships.

### Correlation between DBB and elastic constants

**Figure 6:**
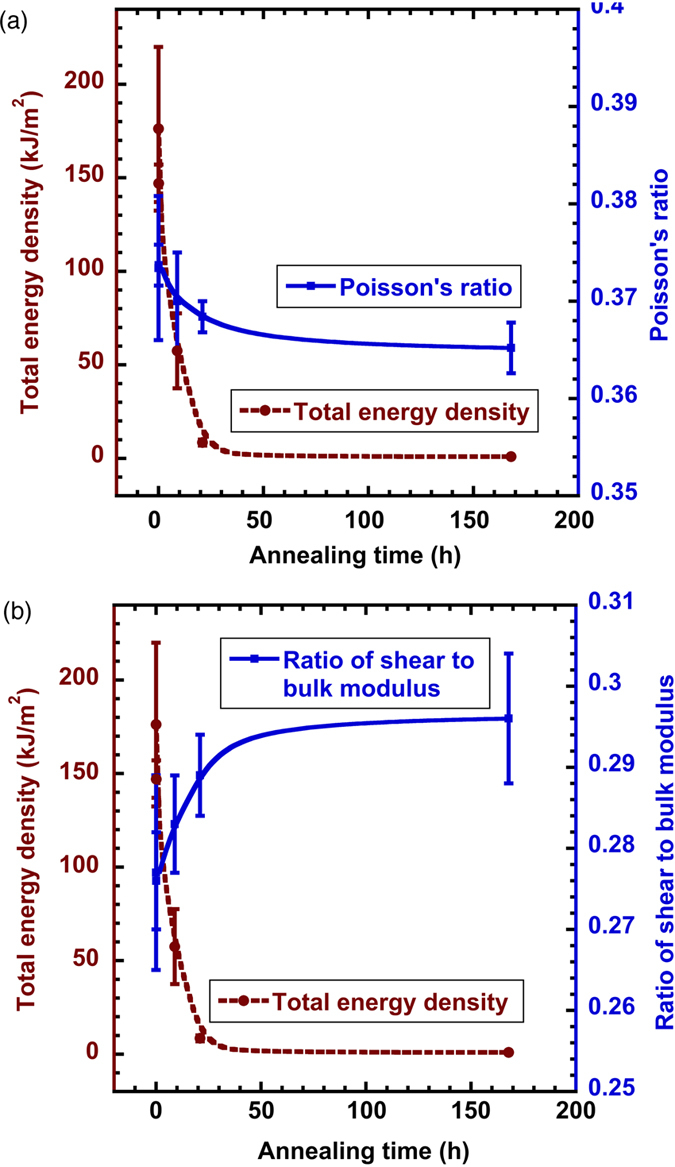
Fracture energy densities of the as-cast and annealed samples, plotted along with the corresponding (a) Poisson’s ratio and (b) shear-to-bulk modulus ratio.

Figure 6 plots the fracture energy density of the as-cast and annealed samples, along with their corresponding elastic properties measured by the ultrasound method. As the decrease of the fracture energy, Poisson’s ratio decrease gradually from 0.373 to 0.365 in Fig. 6(a), and the shear-to-bulk modulus ratio in Fig. 6(b) increases from 0.277 to 0.296. It should be noted that these changes are really small. This basically reflects a tendency that the ductility or fracture toughness of the metallic glasses decreases with the decrease of Poisson’s ratio, or the increase of shear-to-bulk modulus ratio, in accord with previous findings^19^,^20^,^21^,^22^,^23^. However, previous studies suggested a critical value of 0.31 ~ 0.32 for 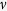 and 0.41 ~ 0.43 for 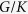, respectively, for the ductile-to-brittle transition^19^. The present work does not agree with their critical values and our Poisson’s ratios only change slightly upon annealing. It is also anticipated that some other types of metallic glasses may have similar variation of fracture energy and elastic constants upon annealing, so that DBB may possibly occur near the starting Poisson’s ratio of the as-cast samples. The correlation between the DBB and a critical Poisson’s ratio of 0.31 ~ 0.32 is clearly of doubts. As stated in the Introduction, treating Poisson’s ratio or the shear-to-bulk modulus ratio as a criterion for determining ductility/brittleness of a metallic glass is empirical and lacks solid physical basis even though it works under many circumstances. The findings here suggest that it is inappropriate to use elastic parameters to judge ductility/brittleness of metallic glasses and a physics-based criterion is needed.

### Structural heterogeneity information extracted from the “mechanical structural probe”

The nanoindentation pop-in experiment is capable of detecting the mechanical heterogeneity in metallic glasses by statistically analyzing variation of the maximum shear stress at the first pop-in, 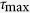, on a relatively large surface area. From nanoindentation pop-in load, the maximum shear stress under the spherical nanoindentation can be calculated by^39^,^47^


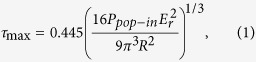


where 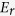 is the reduced modulus given by 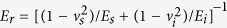 with subscript *s* and *i* indicating the specimen and indenter, respectively. In the present work, 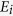 = 1141 GPa and 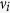 = 0.07 for the diamond indenter, and 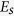 = 89 GPa and 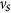 = 0.37 for the Zr-based metallic glass.

**Figure 7:**
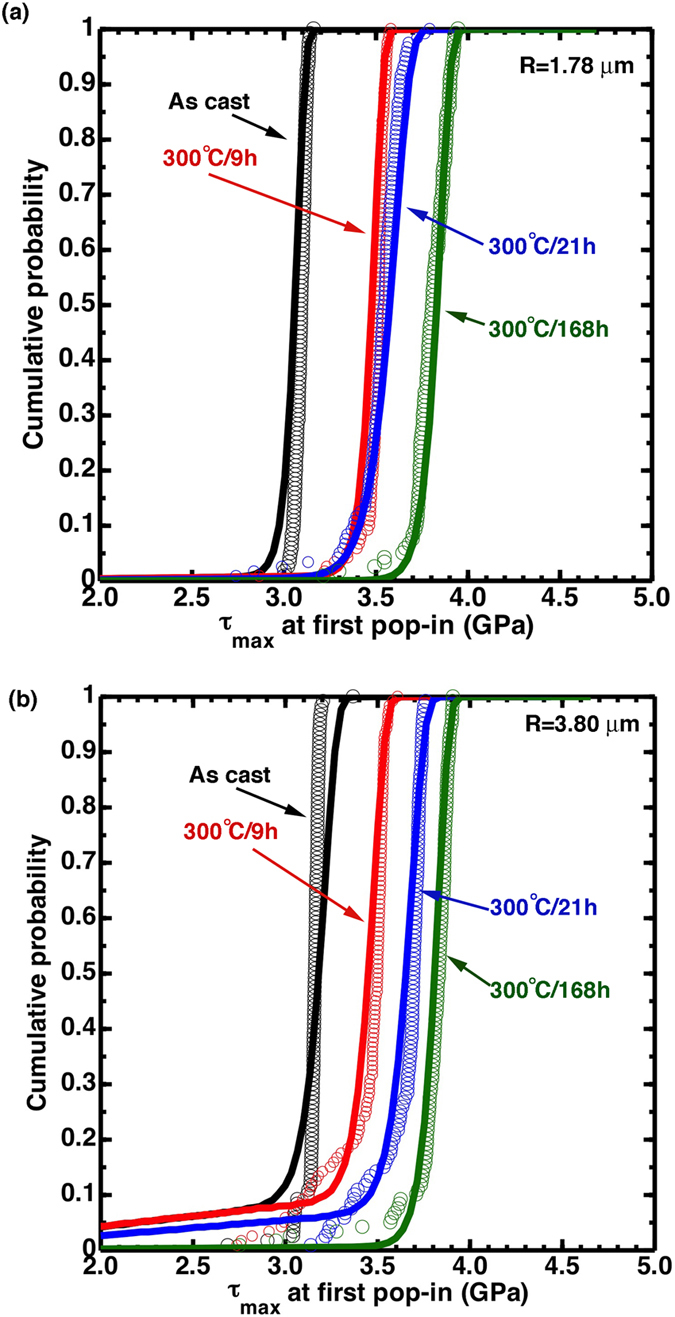
Statistical data of the nanoindentation pop-ins on the as-cast and 300 °C-annealed samples, with spherical indenter tips (a) *R* = 1.78 and (b) 3.80 μm. Symbols indicate experimental data and solid lines are predictions from our unified structural model which incorporates both the thermally-activated shear band nucleation process and defect-assisted shear band initiation process.

Figure 7 shows the obtained statistical pop-in data on the as-cast and 300 °C-annealed samples, using two spherical indenters with tip radii of *R* = 1.78 and 3.80 μm. The cumulative pop-in probability means that the percent of experimental tests that do not see pop-in at a given applied load. With the increase of annealing time, the maximum shear stress at pop-in, 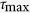, increases and finally reaches a peak value of ~3.8 GPa at 168-hour annealing. We also notice that the as-cast sample and the most structurally relaxed sample (168 hours) have narrow variation in 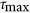, while the two intermediately relaxed samples (9 and 21 hours) have broad distributions and shallower slopes. These trends can be understood from our previously developed structural model for metallic glasses^39^. In this model, structural heterogeneities, which could be defects or soft zones that have a low strength 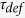, are randomly distributed in a pure-glass matrix that has a theoretical strength 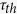. The nanoindentation pop-in tests are essentially a mechanically based “structural probe” that statistically samples these heterogeneities. The as-cast sample has the highest defect density, so the probability of finding these defects in the stressed volume under the indenter is large enough for the pop-in tests to probe the defect strength, 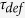, and the variation of the pop-in stress is a result of spatial statistics. After a sufficient period of annealing when the majority of the soft zones are annihilated, the structure is fully relaxed and a “pure fragile glass” state is reached. Under this condition, it is unlikely to find any soft zones under the indenter so that the pop-in stress approaches the theoretical stress, and the variation of the pop-in stress is a result of thermal activation. The transition between these two extreme cases apparently depends on the stressed volume size and the defect density.

We now briefly review the unified structural model^39^, which unifies both the homogeneous shear band nucleation process (thermally activated) and heterogeneous shear band formation process (defect assisted). If the material is in the pure glass sate (fully annealed), the deformation will be completely governed by the thermally activated process. The shear bands can only nucleate when the applied stress reaches the theoretical strength, 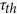, and the cumulative probability of the maximum shear stress is given by


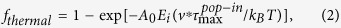


where 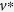 is the activation volume, 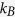 is the Boltzmann constant, *T* is the absolute temperature, 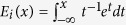 is the exponential integral, 
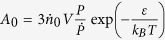
, 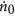 is a pre-factor, 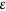 is the intrinsic nucleation energy barrier, and *V* is the stressed material volume. In contrast, in the defect-assisted heterogeneous process, a pop-in event takes place when a pre-existing defect can be found in a sampling volume 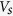 . Following^48^,^49^, the cumulative probability of this defect assisted process can be described by the Poisson distribution


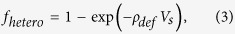


where 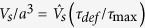, 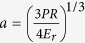 is the contact radius, and this stressed volume can be evaluated from the elastic contact analysis. Consequently, the cumulative pop-in probability in our experiments is a convolution of the above two processes in Eqs. (2) and (3), given by


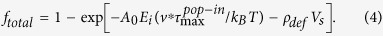


Solid curves in Fig. 7 show prediction with the above unified model. The fitting parameters 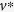 and 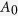 reflect the thermally activated process, and 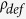 and 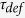 are responsible for the defect-assisted process. The values of fitting parameters under various testing conditions are listed in Table 2. Given that the thermal activation process is not expected to depend on the annealing conditions, 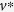 and 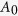 can be obtained from the well-relaxed case (i.e., long time and high temperature annealing). Fitting to all curves give a slight variation of 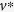 around 0.065, and 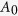 for different conditions within one order of magnitude. The fitting parameter 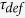 is near the bulk flow stress, so the only fitting parameter that varies significantly with respect to different conditions is the defect density 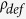. It is found that 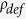 changes substantially as the sample changes from the as-cast sate to the most relaxed sate, i.e. from ~6.0 × 10^15^ m^−3^ to ~0.1 × 10^15^ m^−3^. Clearly, the embrittlement of metallic glasses upon annealing is strongly correlated to the decrease of the pre-existing defects. As expected, the defect strength, 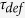, is close to the shear flow strength of these metallic glasses^9^,^47^.

**Figure 8:**
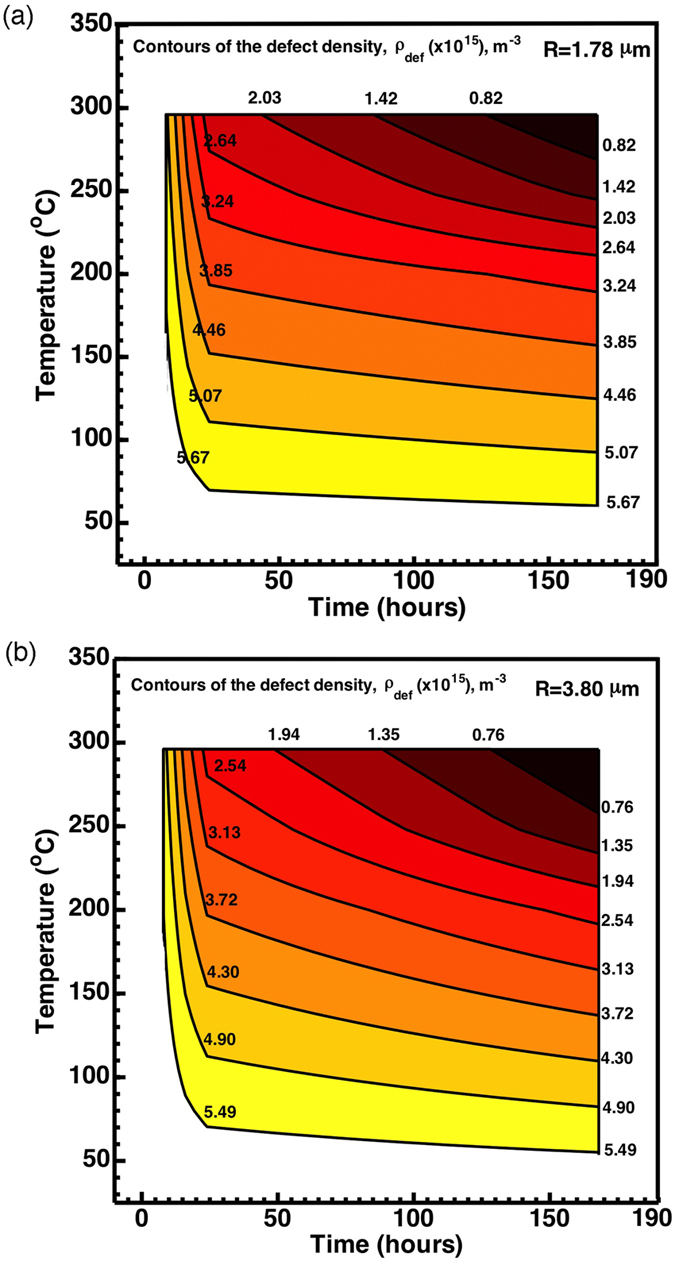
Contours of the calculated defect density from the present work and previous work^39^ with respect to annealing temperature and annealing time for indenter tips of (a) *R* = 1.78 and (b) *R* = 3.80 μm.

Summarizing the results in this work (Table 2) and those from our previous wok^39^, in Fig. 8 we plot the defect density contours with respect to annealing temperature and time for the two indenters with *R* = 1.78 and *R* = 3.80 μm, respectively. The data for samples annealed at 300 °C for varying times in the present work gives the top boundary of the contour plots, the samples annealed at various temperatures for 168 hours in the previous work^39^ sets the right boundary, and the as-cast samples will envelop the left and bottom boundaries. For those unavailable data in between, like 200 °C-9 hour or 250 °C-21 hour annealing conditions, the defect density data are obtained by linear interpolation with respect to the temperature. Contours in Fig. 8 indicate that increasing the annealing temperature and time are two equivalent ways to facilitate embrittlement of the metallic glasses, which is in accord with experimental observations. This demonstrates that the defect density evaluated from our structural model in Eq. (4) is a valid parameter for determining ductility/brittleness of metallic glasses.

The above analysis differs sharply from the previous nanoindentation pop-in studies of metallic glasses^50^,^51^,^52^,^53^,^54^. In the original model by Schuh *et al.*^50^, the pop-in is only governed by a thermal activation mechanism, which leads to two primary fitting parameters: activation enthalpy and activation volume, as in Eq. (2). Pop-in tests on annealed and as-cast samples give different values of these two parameters, which were explained by the presence of soft sites in the metallic glass that indirectly affects the activation volume. In our model in Eq. (4), the thermally activated process is believed to only take place when the theoretical shear strength is approached. A stochastic process is introduced in Eq. (3), which models the spatial statistics in finding pre-existing structural heterogeneity. When interpreting the pop-in measurements using our model, we do not need to adjust the activation enthalpy and activation volume with respect the annealing condition. Rather, the annealing conditions lead to the change of the density of pre-existing structural heterogeneity, as documented in Table 2.

## Discussion

**Figure 9:**
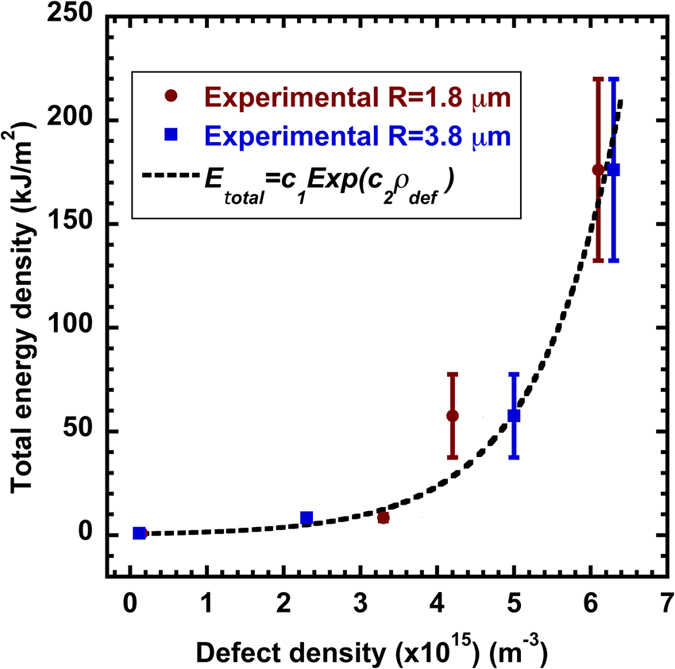
Variation of the total fracture energy density with respect to the defect density for the two indenter tips of (a) *R* = 1.78 and (b) *R* = 3.80 μm. The relationship is described by an exponential function, 
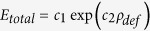
.

Based on experimental data and theoretical modeling in Figs 7 and 8, the total fracture energy density as a function of the defect density for the two different indenter tips is plotted in Fig. 9. Essentially, with the increase of defect density, the fracture energy density first experiences a slight increase followed by a steeper rise. These data can be fitted by an exponential relationship, 
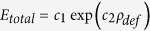
, as shown by the dashed curve in Fig. 9. Values for fitting parameter 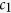 and 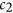 are listed in Table 2. Importantly, this exponential expression allows one to associate the *macroscopic material property* (fracture energy density) with the *microstructural parameter* (defect density) of metallic glasses, which *has never been successfully established* in the study of metallic glasses. One can develop processing methods to vary the internal defect density in order to tune the macroscopic properties such as the DBB.

The structure-property relationship in Figs 8 and 9 also provides an indirect justification of the “immortal” temperature, e.g., 100 °C in our Zr-based metallic glass. Similar to any glassy solid, the relaxation time for Zr-based metallic glass depends critically on the surrounding temperature^43^. One may doubt that annealing for one week at 100 °C may not reach the characteristic relaxation time. The dependence of the fracture energy on the density of structural heterogeneity in Fig. 9, as well as the shallow slopes in the contours in Fig. 8 at low temperature, suggests the slow change of the defect density and thus the persistence of high fracture energy upon annealing at low temperature.

The origin of the ductile-to-brittle transition in crystalline metals varies but is mostly related to dislocation plasticity. For example, at high temperatures, the dislocation can be moved at low flow stresses and a crack tip is hence blunted or blocked by dislocations. In contrast, at low temperatures, the stress required to move a dislocation is high and the material will fail via cleavage fracture. The question that naturally arises is what governs the DBB in metallic glasses.

**Figure 10:**
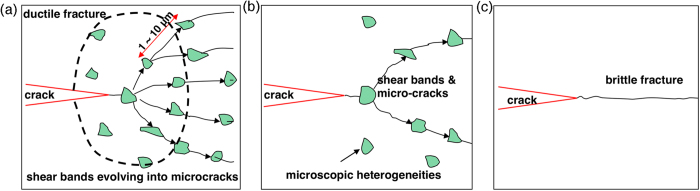
A model of ductile-versus-brittle behavior (DBB), showing different densities of soft zones or pre-existing defects in (a) the as-cast sample, (b) intermediately relaxed sample, and (c) completely relaxed samples. The microscopic heterogeneity density governs the ductile or brittle fracture of metallic glasses.

Based on the results in Figs 8 and 9, here we propose a structural model of the DBB in metallic glasses. The metallic glass consists of the hard matrix and soft zones (i.e., the structural heterogeneities). The hard matrix has glass nature, in which the crack will propagate fast, while the soft zone is more ductile and has capability to blunt a sharp crack. In an as-cast sample, a relatively large amount of soft zones are distributed in the hard matrix, and hence deformation in the vicinity of a crack tip can be easily heterogeneous. The induced plastic deformation by the heterogeneous field tends to blunt the crack tip, and results into ductile behavior as depicted in Fig. 10(a). Annealing the as-cast metallic glass gradually annihilates the soft zones. As the annealing temperature or time increases, more soft zones are annihilated until at a certain critical condition all the soft zones are eliminated and the sample transform to a pure glass sate. In this state, the crack tip would not be blunted because of absence of the heterogeneous deformation field around it, and a sharp crack will propagate through the entire sample as soon as it is initiated as in Fig. 10(c). This will result in nearly-zero macro-plasticity, as happened in the relatively long annealed samples in Fig. 2. In the intermediately relaxed samples, like Fig. 10(b), there are still some soft zones left but not as dense as the as-cast state. Accordingly, the effectiveness for preventing brittle failure of the metallic glasses is not as good as that in the as-cast state, resulting in limited ductility. This corresponds to those samples having intermediate energy density values in Fig. 2.

In comparison, Murali *et al.*^35^,^36^ develops an atomic-level description of the brittle/ductile fracture. That is, the brittle fracture is governed by a cavitation mechanism via multiple nanoscale void nucleation and coalescence in the front of the crack tip, as opposed to blunting of the crack tip through extensive shear banding behavior in the ductile fracture. Our work gives a scenario of the brittle versus ductile behavior at the microscopic level, while theirs is on nanoscopic scale. The counterparts in crystalline materials are the competition between dislocation emission and atomic cleavage for Murali *et al.*^35^,^36^, and the competition between crack tip process zone (such as plasticity, micro-damage, crack bridging zone in composites, etc.) and cleavage fracture for our work here. These two models clearly complement to each other, and one can develop a constitutive model of the shear banding behavior by incorporating their nanoscale cavitation mechanism, and simulate the roles of microstructural heterogeneity on the crack tip process zone that provides the resistance to the crack propagation.

## Summary and Conclusions

Through annealing the metallic glasses at different temperatures for various times, the ductile-versus-brittle behavior of these materials is systematically studied. The physical causes responsible for the DBB were investigated by various techniques that measure thermal, mechanical, and structural properties. The following conclusions can be drawn.

(1) Embrittlement upon annealing occurs only above a critical temperature. Below this critical temperature, the metallic glass is not expected to become brittle regardless of the annealing time. This temperature is ~100 °C in the present work for a Zr-based metallic glass.

(2) Increasing the annealing temperature or time are two equivalent ways to transform the metallic glass from the ductile to brittle state. When processed at high temperatures or long annealing time, the metallic glass will behave as a “fragile glass”; the fracture behavior is similar to silicate glasses with mirror finish fracture surface and the fracture energy approaches zero from our three-point bending tests.

(3) The differential scanning calorimetry did not find any significant changes in crystallization temperature and enthalpy, indicating that the materials still remained fully amorphous. However, a slight increase in *T*_*g*_, or decrease in *∆T*_*x*_ in annealed samples, or shape changes in the glass transition peaks do reflect a certain degree of structural relaxation upon annealing.

(4) Poisson’s ratio is found to show a decreasing trend as the metallic glass become brittle by annealing, while the shear-to-bulk modulus ratio displays an increasing trend. The findings do not agree with the previously proposed critical value that determines brittleness or ductility of a given metallic glass.

(5) By performing statistical nanoindentation pop-in tests that provide a mechanically-based “structural probe”, the defect density is found to be a more effective parameter for determining the ductility/brittleness of metallic gasses. Our unified structural model nicely describes the increasing, but narrowly distributed, nanoindentation pop-in loads as these metallic glass samples are being annealed, which was interpreted as a result of gradual annihilation of these structural defects. The compilation of experiments under various annealing conditions allows us to construct a defect density-temperature-time map.

(6) A DBB model with regard to the essential physical processes is developed. When the sample is as-cast or not fully annealed, there are finite amounts of structural heterogeneities (defects or soft zones) and hence the material exhibits certain degrees of ductility. As the material is completely annealed to the pure glass state by exposing to high temperatures or long annealing times, the defects are nearly completely annihilated and the brittle behavior is encountered.

(7) The microstructural parameter (defect density) is directly linked to the macroscopic fracture energy density by an exponential relationship. This opens opportunities to tune macroscopic material properties from microstructural means.

## Methods

Five elemental constituents, Zr (99.5%), Cu (99.99%), Ni (99.99%), Al (99.99%) and Ti (99.99%), were mixed in appropriate proportion and arc melted in Ti-gettered Argon atmosphere to first prepare the metallic glass buttons. For uniform mixture of constituents, buttons were flipped and remelted 5 ~ 6 times. Zr_52.5_Cu_17.9_Ni_14.6_Al_10_Ti_5_ metallic glass (BAM 11) rods with a diameter of ~7 mm and length of ~75 mm were subsequently fabricated by arc melting buttons in Helium atmosphere and dropping into a water-cooled cylindrical copper mold, which were marked as the as-cast samples. Rectangular specimens with a dimension of about 2.5 × 2.5 × 15 mm were cut from central part of the metallic glass rods using electric discharge machining, and notches of about 0.3 × 0.3 × 2.5 mm were cut out in the middle section of each individual sample. Specimens were finely ground to remove the oxidation layers created by cutting and subsequently annealed in vacuum for 10 mins, 9 hours, 21 hours and 1 week at 100 ~ 300 °C, which are well below the glass transition temperature, *T*_*g*_ ≈ 400 °C. Single-edge notched three-point bending tests were performed at quasi-static condition. Fracture tests were carried out at room temperature on Instron 5881 screw-driven universal mechanical testing system with a strain rate of 2.5 × 10^−3^ s^−1^. At least three samples were repeated at each condition. Fracture surfaces were examined by the scanning electron microscopy (SEM).

The differential scanning calorimetry (DSC) was used to investigate thermal properties of the as-cast and annealed samples, e.g., glass transition temperature, crystallization temperature, supercooled liquid region, and others. DSC was run at a constant heating or cooling rate of 20 K/s under a purified argon gas flow. Two cycles of heating and cooling were performed, i.e. the samples were heated up till completion of the crystallization followed by a complete cooling down to ~200 °C, and then the same procedure was repeated in the second cycle. The second cycle is served as a baseline in analysis. The glass transition temperature 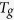, crystallization temperature 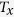, and enthalpy of crystallization 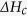 are analyzed by the *Proteus* thermal analysis software.

Elastic properties were measured with the ultrasonic technique^55^. Owing to the material isotropy, only two independent elastic parameters, Young’s modulus and Poisson’s ratio, need to be determined. This was accomplished via measurement of the longitudinal and transverse wave velocity, 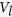 and 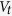. The Young’s modulus, *E*, and Poisson’s ratio, 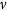, are then calculated by


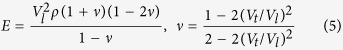


where 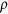 is the material density obtained by measuring the mass and volume of the sample. The mass and volume were measured by an AccuPyc^TM^ 1330 pycnometer with a precision of 0.0001 g and 0.03%, respectively, giving rise to a density accuracy of better than 0.01g/cm^3^.

Specimens to be indented, including the as-cast and annealed samples, were mounted in the epoxy resin, then ground and polished to eliminate all the ground damage which ensures that pop-ins can be observed during nanoindentation. Spherical nanoindentation with indenter tip radius of 1.78 and 3.80 μm (calibrated using the method in ref. 56) was performed on a Nanoindenter-XP system in continuous stiffness mode (CSM) with a constant loading rate 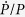 = 0.05 s^−1^. Around 121 indents were made on each sample for statistical analysis, and indents were placed far enough to avoid interference. Evident pop-ins, i.e., sudden discontinuous excursion on the load-displacement curves, were observed on almost all indented specimens, and corresponding loads at pop-ins were termed as pop-in loads (*P*_pop-in_).

## Additional Information

**How to cite this article**: Li, W. *et al.* On the correlation between microscopic structural heterogeneity and embrittlement behavior in metallic glasses. *Sci. Rep.* **5**, 14786; doi: 10.1038/srep14786 (2015).

## Figures and Tables

**Table 1:** Thermal properties obtained from the differential scanning calorimetry (DSC), including the glass transition temperature (*T*_*g*_), onset crystallization temperature (*T*_*x*_), supercooled liquid region (Δ*T*_*x*_), and the enthalpy of crystallization (Δ*H*_*c*_).

**Sample**	***T*_*g*_ (°C)**	***T*_*x*_ (°C)**	***∆T*_*x*_ (°C)**	***∆H*_*c*_, J/g**
As-cast	398.0	459.0	61.0	−48.84
Annealed (300 °C, 9 hrs)	406.0	458.5	52.5	−49.75
Annealed (300 °C, 168 hrs)	405.0	459.0	54.0	−48.96

**Table 2:** Fitting parameters for the unified structural model that describes the nanoindentation pop-ins, as well as the exponential functions that relate the total fracture energy density to the defect density.

**Indenter radius R, μm**	**1.78**				**3.80**			
Annealing time, hours	As-cast	9	21	168	As-cast	9	21	168
v^*^, nm^3^	0.071	0.072	0.063	0.066	0.068	0.067	0.071	0.065
*A*_*0*_ (×10^−22^)	2.03	3.00	1.17	6.93	4.90	1.89	1.92	5.36
*τ*_def_, GPa	1.11	1.14	1.14	1.08	0.98	0.95	1.11	0.98
*ρ*_def_ ,×10^15^ m^−3^	6.30	5.00	2.30	0.12	6.10	4.20	3.30	0.15
*c*_1_, kJ/m^2^	0.83							
*c*_2_,×10^−15^ m^3^	0.88							
